# User Requirements for an Electronic Patient Recruitment System: Semistructured Interview Analysis After First Implementation in 3 German University Hospitals

**DOI:** 10.2196/56872

**Published:** 2024-09-27

**Authors:** Alexandra Stein, Romina Blasini, Cosima Strantz, Kai Fitzer, Christian Gulden, Torsten Leddig, Wolfgang Hoffmann

**Affiliations:** 1 Institute for Community Medicine Section Epidemiology of Health Care and Community Health University Medicine Greifswald Greifswald Germany; 2 Institute of Medical Informatics Justus Liebig University Giessen Germany; 3 Medical Informatics Institute for Medical Informatics, Biometrics and Epidemiology Friedrich-Alexander Universität Erlangen-Nürnberg Erlangen Germany; 4 Core Unit Data Integration Center University Medicine Greifswald Greifswald Germany

**Keywords:** patient recruitment system, clinical trial recruitment support system, clinical trials, recruit, recruitment, recruiting, participant, participants, research, digital health, usability, interview, interviews, qualitative, experience, experiences, attitude, attitudes, opinion, perception, perceptions, perspective, perspectives, database, databases, information system, information systems, search, searches, searching, retrieval

## Abstract

**Background:**

Clinical trials are essential for medical research and medical progress. Nevertheless, trials often fail to reach their recruitment goals. Patient recruitment systems aim to support clinical trials by providing an automated search for eligible patients in the databases of health care institutions like university hospitals. To integrate patient recruitment systems into existing workflows, previous works have assessed user requirements for these tools. In this study, we tested patient recruitment systems KAS+ and recruIT as part of the MIRACUM (Medical Informatics in Research and Care in University Medicine) project.

**Objective:**

Our goal was to investigate whether and to what extent the 2 different evaluated tools can meet the requirements resulting from the first requirements analysis, which was performed in 2018-2019. A user survey was conducted to determine whether the tools are usable in practice and helpful for the trial staff. Furthermore, we investigated whether the test phase revealed further requirements for recruitment tools that were not considered in the first place.

**Methods:**

We performed semistructured interviews with 10 participants in 3 German university hospitals who used the patient recruitment tools KAS+ or recruIT for at least 1 month with currently recruiting trials. Thereafter, the interviews were transcribed and analyzed by Meyring method. The identified statements of the interviewees were categorized into 5 groups of requirements and sorted by their frequency.

**Results:**

The evaluated recruIT and KAS+ tools fulfilled 7 and 11 requirements of the 12 previously identified requirements, respectively. The interviewed participants mentioned the need for different notification schedules, integration into their workflow, different patient characteristics, and pseudonymized screening lists. This resulted in a list of new requirements for the implementation or enhancement of patient recruitment systems.

**Conclusions:**

Trial staff report a huge need of support for the identification of eligible trial participants. Moreover, the workflows in patient recruitment differ across trials. For better suitability of the recruitment systems in the workflow of different kinds of trials, we recommend the implementation of an adjustable notification schedule for screening lists, a detailed workflow analysis, broad patient filtering options, and the display of all information needed to identify the persons on the list. Despite criticisms, all participants confirmed to use the patient recruitment systems again.

## Introduction

### Background

Clinical trials are the gold standard of evidence-based medicine and are indispensable for medical progress. New diagnostics, therapies, and medications usually need to be evaluated in a randomized clinical trial. Despite the importance of clinical trials, it is often difficult for trial staff to identify a sufficient number of patients who meet the specific eligibility criteria of clinical trials and who are willing to participate. Therefore, many trials fail to include enough patients, thereby leading to statistical and financial as well as ethical problems in medical research [1-3]. One reason for this is the lack of time capacity of the trial staff [2].

Electronic systems can help to identify potential trial participants in hospitals or other health care institutions by generating a screening list of all patients who fulfill the eligibility criteria [4-6]. For example, in 2015, McCowan et al [7] published a report on stakeholders from various countries in Europe for the project EHR4CR (electronic health records systems for clinical research), which aimed to enhance the utilization of electronic health records for clinical research. Their findings indicated that a significant proportion of stakeholders perceived that a platform could facilitate the implementation of clinical trials [7].

Most of the described patient recruitment systems (PRSs) were implemented for a specific site or trial. The PRS approach is time-consuming and costly and therefore not scalable for other trials [8,9]. Few systems have been built with a generic approach, independent of specific use cases to support a wide range of experiments [5].

### Medical Informatics in Research and Care in University Medicine

Data integration centers were established at university hospitals as part of the MIRACUM (Medical Informatics in Research and Care in University Medicine) project, a large-scale initiative in German medical informatics focusing on research and care in university medicine. One part of MIRACUM was the so-called Use Case 1 (alerting in care), which aimed to develop and evaluate a hospital-wide PRS in a multicentric study across all participating sites. The implemented systems, namely, recruIT and KAS+, were evaluated in this feedback analysis. Both systems are briefly presented in the following paragraphs.

**Figure 1:**
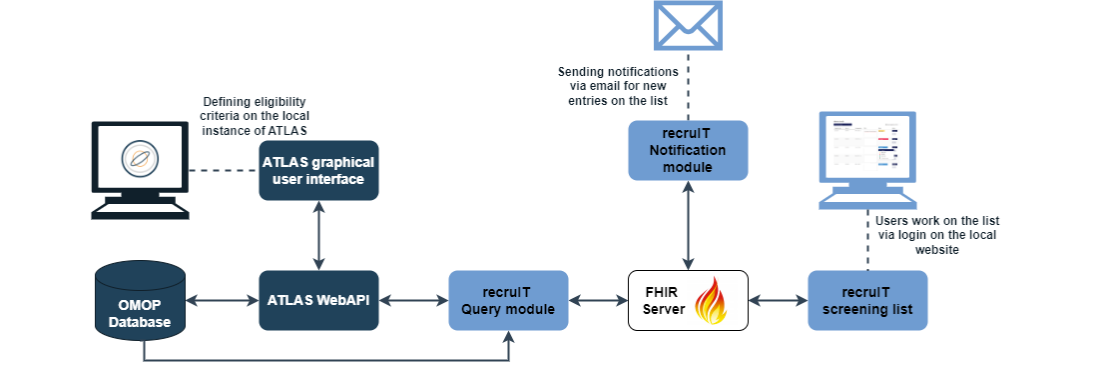
Architecture of the recruIT system. recruIT components are displayed in light blue, and Observational Health Data Sciences and Informatics (OHDSI) components are displayed in dark blue. The eligibility criteria are portrayed with the ATLAS graphical user interface. The query module triggers the search for new patients and writes all the results in the central Fast Healthcare Interoperability Resources (FHIR) store. The graphical user interface of recruIT (screening list) displays the results as a website. Users are informed about new results via email. API: application programming interface; FHIR: Fast Healthcare Interoperability Resources; OMOP: Observational Medical Outcomes Partnership.

As part of the MIRACUM project, a recruitment system has been in place at several sites to support a wide range of trials [10]. Based on previously identified system requirements [11], the software recruIT (MIRACUM project) was developed. The system is shown in Figure 1 and described in detail in [11]. This system relies on the Observational Medical Outcomes Partnership (OMOP) common data model, which is a software tool of the Observational Health Data Sciences and Informatics (OHDSI) [12]. The eligibility criteria of the trials can be formulated using the ATLAS (OHDSI) software. RecruIT generates a list of potentially eligible patients, which can be accessed through an internal website that shows all the basic information such as patient number, age, and gender of all entries [5].

**Figure 2:**
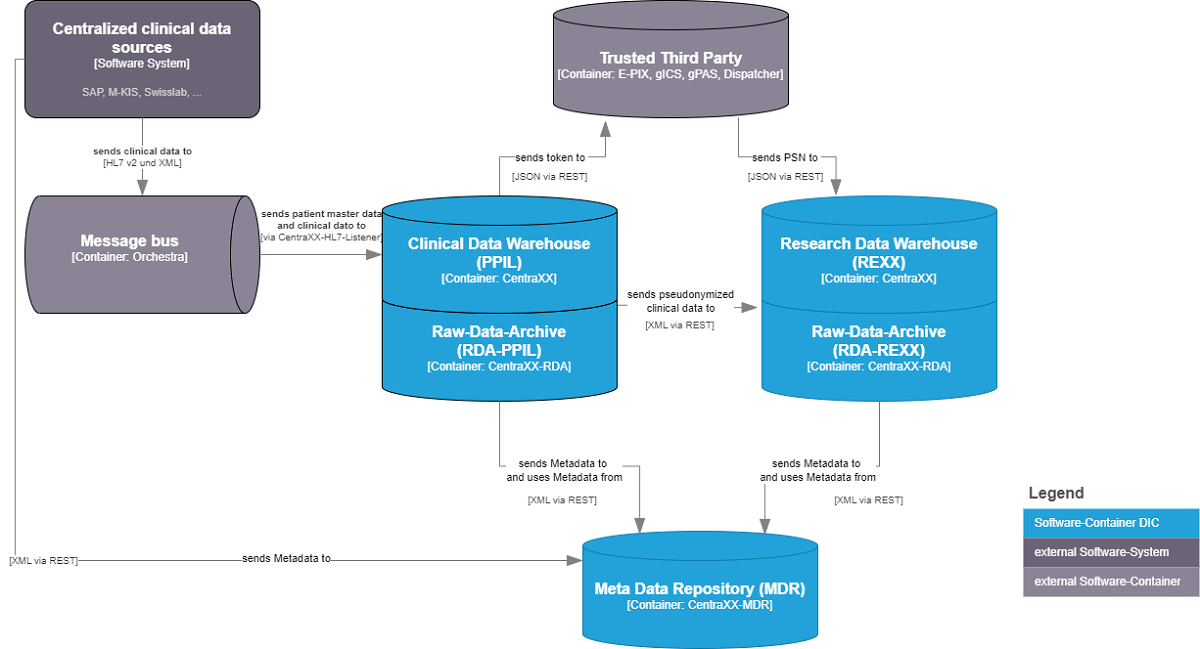
Architecture of the KAS+ system. Data integration center components are shown in light blue, and external components are shown in gray. Eligibility criteria are managed in the CentraXX instance called Privacy Protection and Interface Layer. The CentraXX query module initiates the search for new patients, writes all the results to its internal database, and sends proposals to the hospital information system. PPIL: Privacy Protection and Interface Layer.

Within the KAS+ infrastructure (Figure 2), all clinical systems transmit the patient data via HL7v2 and XML to the communication server orchestra. This distributes the data between the clinical systems and immediately transfers the data to the research platform. This consists of 2 CentraXX instances and 2 CentraXX raw-data-archives. The clinical data are read into Privacy Protection and Interface Layer, and if informed consent has been given, the data are pseudonymized using the trusted third party tools and transferred to REXX. Within the research platform, the trials are administered and the inclusion and exclusion criteria are defined. If it is configured for a defined study, CentraXX immediately checks any new patient’s data to determine whether a patient may be eligible for a trial and sends the proposal to the hospital information system (HIS).

### Requirements of PRS

In order for the system to be useful to the trial staff and clinicians, it needs to be fully integrated into their workflow [13]. Research has been conducted on the topic of implementing and evaluating PRS and on data elements needed for that purpose [11,14,15]. For example, Schreiweis and Bergh [14] performed unstructured interviews and identified PRS requirements of different health care actors. Although Schreiweis and Bergh [14] described the fundamental prerequisites, the specific desires of researchers for a PRS with integration in diverse workflows remain largely unidentified. Aside from the capacity to search for eligible individuals with the assistance of software, there is a paucity of information regarding the specific requirements researchers have for a PRS [14]. In a previous work [11], a number of people involved in patient recruitment were interviewed to assess how the recruitment process currently works, which data sources are useful, and which features they need from a PRS in general. With this information, a list of requirements was developed that a PRS should fulfill in order to meet the requirements of the trial staff.

### Objective

The goal of our work is to investigate whether and to what extent the tools of the MIRACUM project can fulfill the requirements resulting from the initial requirements analysis. Feedback should come from the real-world environment of patient recruitment. Therefore, a test phase is needed in which the trial staff will use the tool in their day-to-day work. A user survey will be conducted to determine whether the tools are usable in practice and helpful for the trial staff. The survey can also show whether additional requirements might arise from the test phase that were not considered in the initial requirements analysis. To avoid misunderstandings, we will refer to studies using the PRS as “trials” and to the investigation described here as our “study.”

## Methods

### General Procedure

We conducted semistructured interviews with users of recruIT and KAS+ and derived requirements and feedback on the systems from these. Users (trial staff) had access to PRS instruments for at least 1 month. After this testing period, interviews were conducted according to the interview guide.

**Figure 3:**
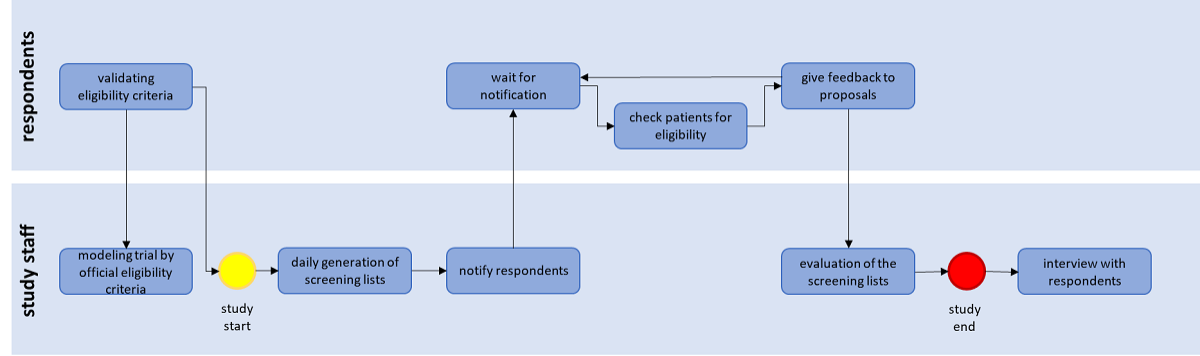
Processes of the study involving users and study staff.

The test phase was part of an evaluation study to review the effectiveness of the software tools at 7 university hospitals. More detailed information regarding this study can be found in [16]. Figure 3 shows the tasks the study staff and the respondents had during the study.

All respondents supervised at least 1 trial during the testing phase. For this study, 3 university hospitals from the evaluation study were included with all the respondents who gave interviews for analysis, either recorded and transcribed or stenographed. The other 4 university hospitals could not provide recorded or transcribed interviews, which is why they could not be included in this evaluation.

A few eligibility criteria were given in the study itself, such as the exclusion of trials with focus on psychological diagnosis. Other criteria were established by the sites to consider the local particularities: the exclusion of trials regarding children or cancer diagnosis, as the size of that site did not make such a tool necessary because the staff know the suitable patients. Another criterion for the trial selection was the expected recruitment of at least 4 patients over the course of 1 year to generate analyzable data.

The interview partners were selected at the respective study locations by the primary investigators. This approach was designed to leverage the domain knowledge and the local networks of the investigators to recruit test individuals for the study in an optimal manner. Potential individuals were invited to participate in this study, and if they consented, a time was arranged for a face-to-face interview.

### PRS

**Figure 4:**
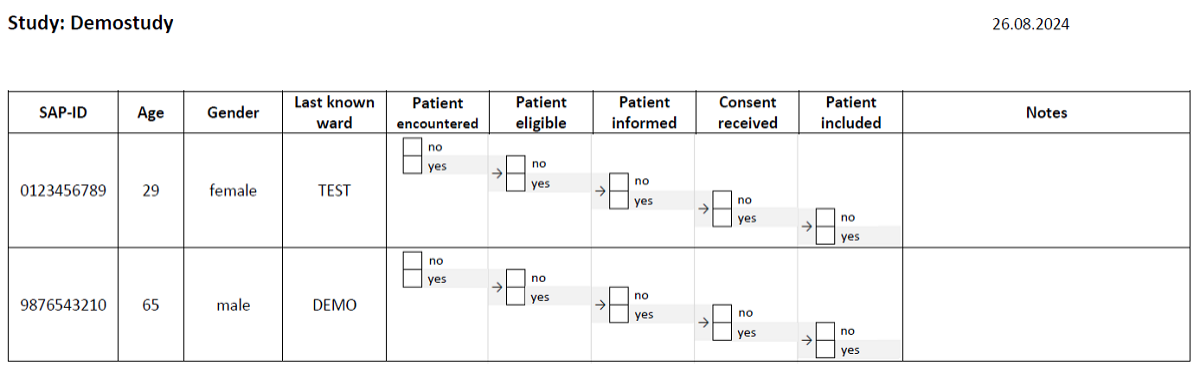
A mock screening list of the CentraXX system, which is provided to trial personnel in the form of a PDF file.

From KAS+, only part of the PRS was used during the study to meet the requirements of the ethics committee and generate the feedback necessary for the evaluation. We used the search engine in CentraXX, and the parameters used were defined together with the trial personnel. From the search results generated by CentraXX, we created the so-called screening lists with an SQL query. These results were copied to a template with feedback options. An example list is shown in Figure 4. Each morning, participants received an email with the screening list if any potentially eligible patients were identified or a notice that no suggestions had been generated. These lists were then used by the trial staff according to their usual recruitment workflows. The tabular format provides information on age, gender, and the last ward stored in the system for each patient ID. The adjacent checkboxes are used to record the recruitment status. They are also required to record the feedback on the proposals necessary for the study.

**Figure 5:**
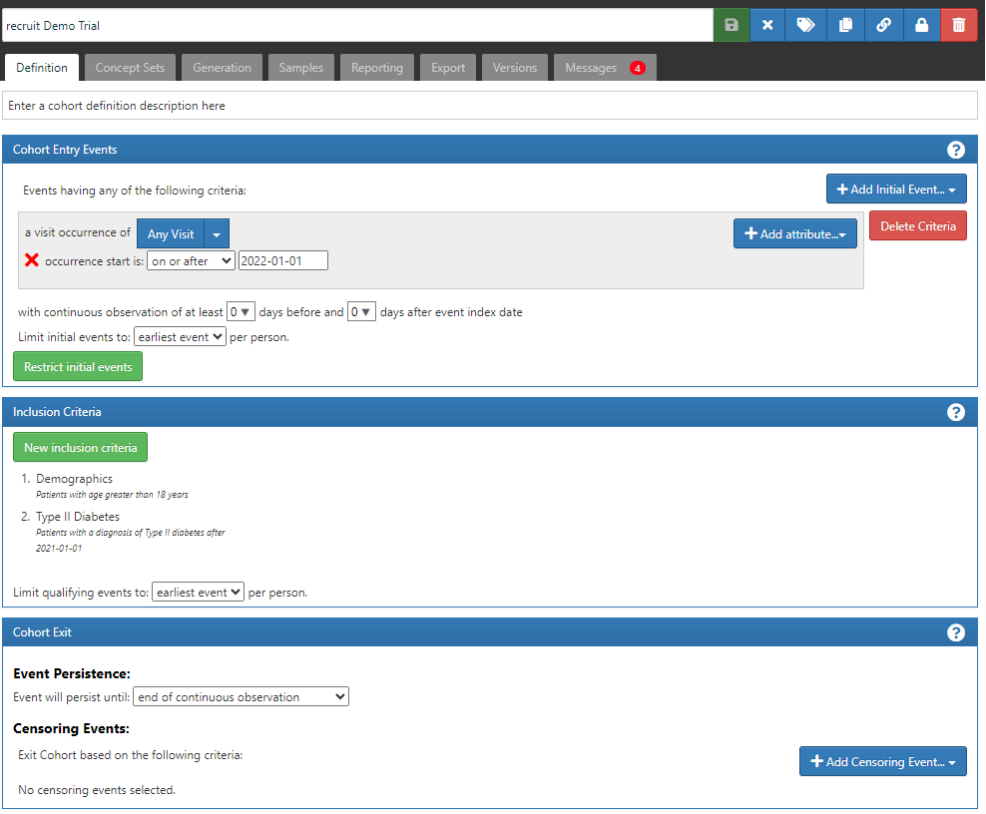
Sample trial as represented in the ATLAS software. All eligibility criteria are defined under inclusion criteria. In this example, the trial is looking for people who have been hospitalized since 2022, have type 2 diabetes, and are older than 50 years.

Of the 3 sites included, 2 utilized the recruIT system to generate screening lists. The initial step in utilizing the system was to translate the eligibility criteria of all the participating trials to ATLAS cohorts. In the OMOP common data model, all information is represented by a medical terminology system. Consequently, we also identified the corresponding codes and units of the aforementioned terminology systems for each eligibility criterion, prior to their portrayal in ATLAS. This procedure is also described in [17]. Figure 5 illustrates a cohort definition in ATLAS for 1 trial.

**Figure 6:**
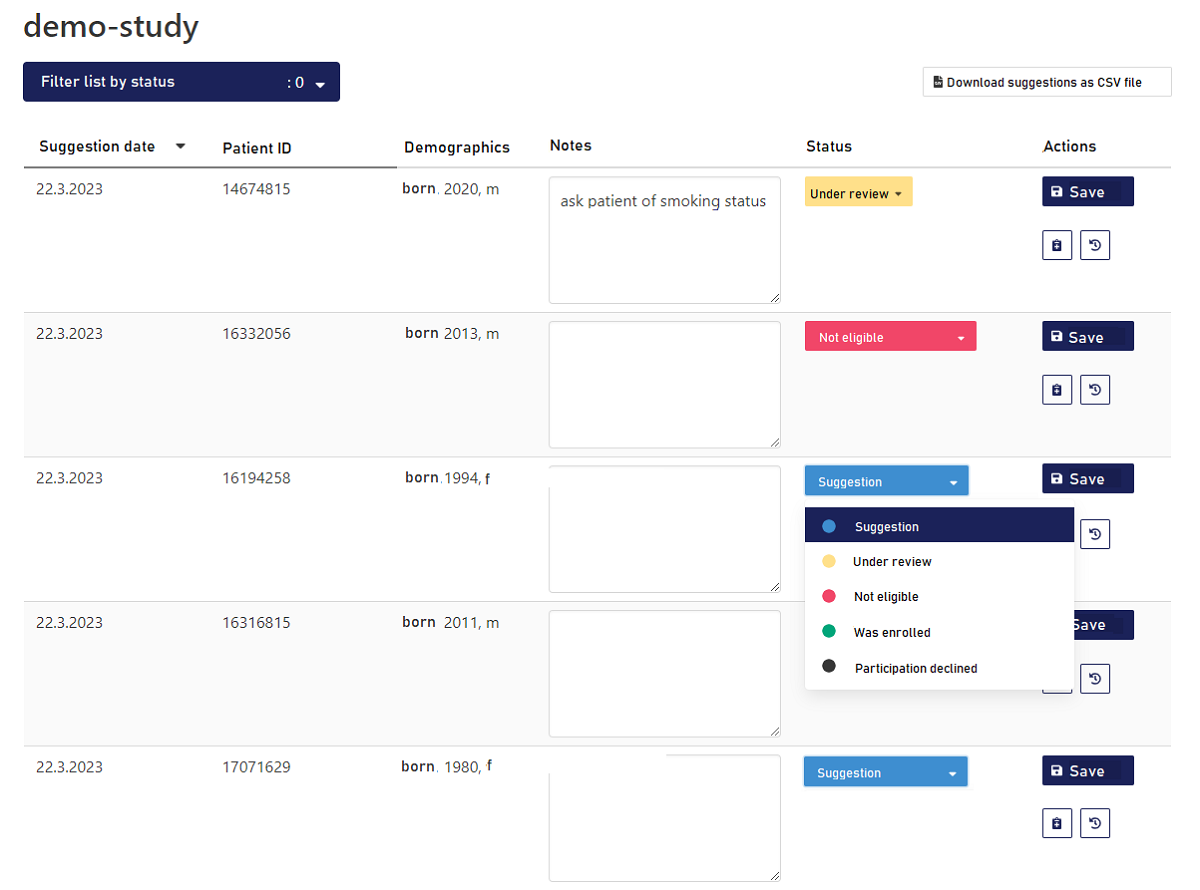
Exemplar representation of the screening list of the recruIT system. The original screenshot was overwritten with English translation.

Both sites used individual configurations in accordance with local ethics committee recommendations and data protection regulations. This leads to different information shown on the web-based screening list, which is shown in Figure 6. In both sites, a patient identification number was displayed as well as the date of the first suggestion of the patient and the recruitment status. The latter can be updated by the trial staff, and a text box is provided for each entry to store additional free text regarding the proposal. Additionally, the list shows when a patient is not eligible anymore, for example, when he/she is discharged from the hospital or in case that the patient has been enrolled in another trial. For 1 site, some more information about the patients was shown on the list. This included gender, birth year, and information about the last visit and ward. The systems were updated on a daily basis, and notifications were configured either daily, weekly, or several times a week, in accordance with the user’s wishes.

### Fulfillment of Requirements

Identifying the requirements met by the tools is the first step. For this purpose, the results of Fitzer et al [11] were used, and each requirement was compared with the functional scope. These requirements were extracted and compiled into a table. Subsequently, it was indicated for both tools whether they completely fulfill, partially fulfill, or do not fulfill these requirements at all. For the KAS+ site, both the versions used in the study context and HIS integration were assessed.

### Interviews

The authors used semistructured interviews. Most of the questions were open-ended. These questions asked interviewees to describe the process of identifying eligible patients with and without PRS. Additional questions were included, that is, if there were problems with usability and whether the system could be integrated into their workflow, with room for additional statements about their experiences. Moreover, we added 2 questions that required only a yes or no answer: whether the system could be integrated into their workflow and whether they would use it again. In addition, we asked for some demographic data, which include age and experience with patient recruiting. The full list of questions and their order is shown in Multimedia Appendix 1. Although some of the questions required a “yes” or “no” answer, participants were given the opportunity to provide more detailed responses in full text, and if they did, we included those responses as well in our analysis.

For organizational reasons, 1 site asked additional questions as described in the last part of the table in Multimedia Appendix 1. Once all the interviews were transcribed, they were independently coded by 2 authors (RB and AS) according to Meyring method [18,19]. In this approach, the text to be analyzed was first examined for its key statements, and these were then summarized. These statements were then generalized into codes, and codes with the same meaning were summarized. The generalization was performed on the basis of a previously defined category system, which was then checked again against the source material [18,19]. After categorizing the codes, we structured and sorted them by using categories. Furthermore, any statements that contained a requirement for recruitment tools were marked. Afterward, the responses from the interviews were compared with the requirements already identified in [11] and checked to see if they were the same or if new ones had been mentioned.

### Ethics Approval

This study received institutional review board approval from the ethics committees of Friedrich-Alexander University Erlangen-Nuremberg (approval 89_20B) as well as of Justus Liebig University Giessen (approval AZ 193/20) and Greifswald University Medicine (approval BB 084/20). Within this study, no identifying personal data were centrally collected and analyzed. No compensation was offered.

## Results

### Study Participants

This study consists of a total of 11 participants, comprising 7 clinical trial investigators, 2 research assistants, and 2 physicians. In 1 instance, the interview was conducted with 2 individuals simultaneously. The ages of the interviewees ranged from 25 to 34 years (2 participants), 35 to 44 years (4 participants), and 45 to 54 years (4 participants). The average number of years of professional experience in patient recruitment was 10.4 years (range 1-21 years). Four participants worked in the field of neurology, 2 in cardiology, and 1 person each in the fields of dermatology, internal medicine, neurosurgery, and rheumatology. The participants used the screening list for 1-3 trials each.

### Degree of Compliance With Requirements

In [11], the following 6 categories of requirements are described: notifications, overview of patients, overview of trials, search, patient data, and user management and interface. We omitted the category “overview of trials” in this study since it is implemented as part of another tool at all participating sites. The category “patient data” contains data elements that can be used for searching; all other categories are shown in Table 1. Both systems fulfill the main requirement of (1) generating a list of eligible patients and (2) notifying users. In addition, it is possible to tag participants, make notes, and track the recruitment status. Both investigated systems lacked integration with existing HISs. Comparison with clinical trial eligibility criteria is possible with diagnoses, demographics, laboratory results, and vital signs in both tools. The treatment data mentioned in [11] can only be partially queried by the tools.

**Table 1:** List of requirements defined by Fitzer et al [11] and implementation in the recruIT and KAS+ systems. Since a KAS+ test environment with different properties was used for this study, this is also indicated.

Requirements			recruIT (n=12)		KAS+ evaluation environment (n=12)		KAS+ (n=12)
**Notifications**							
	Users are instantly notified if new suggestions are available	Yes^a^		Yes		Yes	
	Notifications are adjustable to individual preferences by the user	Yes		No^b^		No	
**Overview of patients**							
	Supports a list of all patient suggestions	Yes		Yes		Yes	
	Possibility to check suggestions by themselves	Yes		No		Yes	
	The list with suggestions is integrated into existing systems	No		No		Yes	
	Option to mark participants	Yes		Yes		Yes	
	Option to make notes	Yes		Yes		Yes	
	Option to track the recruitment status	Yes		No		Yes	
	Edit recruitment list by manually adding patients to list	No		No		Yes	
	Edit recruitment list by removing patients from list	Yes		No			
	Integrating patient summaries into the patient recruitment system	Yes		No		Yes	
**Search**							
	Offers sophisticated search options	Partially^c^		Yes		Yes	
**User management and interface**							
	Contain a sophisticated rights concept to account for the various roles in the trial and at the clinical center	Partially		No		Yes	
Requirements fulfilled			8		5		11

^a^Yes: implemented.

^b^No: not implemented.

^c^Partially: partially implemented.

### Interview Results

As the participants had no restrictions on how to integrate the tool into their workflow, the kind of integration varied. For 3 participating trials, the tool was a permanent part of the workflow; for others, it was used when the staff had spare time (n=2). Two interviewees made it the preferred source for patient recruitment. When analyzing the transcribed interviews, we found 54 different codes. To put the codes into context with each other, we defined 5 groups: system integration, parameter, precision, system evaluation, and user interface.

#### System Integration

Statements about the update frequency differed between the requirement to enable real-time recruitment, daily, and weekly updates. The requirement to flexibly adjust the update and notification interval per trial was also mentioned by 3 participants. All other statements required a more flexible integration into the daily workflow of the trial staff. The lists should be processed flexibly, when there is time in the clinical daily work, and the list should be integrated into the local HIS. Generally, there should be no system discontinuities.

#### Parameter

Three respondents indicated that not all relevant criteria were available in the system, and 9 respondents mentioned specific data elements, namely, medications, pulmonary parameters, lung transplant list, laboratory results, cardiac echocardiography findings, general findings, admission letters, and alcohol abuse. In addition, 6 respondents expressed the necessity of filtering the list by ward, while 1 respondent proposed that this should encompass the entire patient journey. One respondent cited poor data quality and inadequate utilization of the International Statistical Classification of Diseases (ICD) and Related Health Problems for all criteria related to diagnosis (n=3).

#### User Interface

The majority of the statements in this group pertain to patient identification. Patient numbers should be fully displayed, and pseudonymous lists are considered impractical to use. In 1 interview, the full name of each patient was also requested, while in another one, this was mentioned as not relevant. The new features cited included more options for patient recruitment status (n=1), integration of better categorization and tagging options in the list (n=1), and the ability to sort suggestions by ward (n=1). In addition, 1 person commented that it would be nice to add a third category of soft exclusion criteria that would result in a warning on the generated list. This would be especially helpful in the case of discretionary decisions, for example, if patients are excluded due to a certain diagnosis, all patients on the list who had this diagnosis in the past should be flagged on the list so that the trial staff know that they need to check whether this diagnosis is still valid.

#### Precision

It was mentioned that the list contains too many suggestions that are not eligible for the trial (n=5) as well as too many eligible persons that are not on the list (n=2). The list contained no or few false positives (n=3) or false negatives (n=1). In addition, 1 respondent mentioned that subsequent adjustments to the filter criteria resulted in more accurate screening lists.

#### System Evaluation

The results regarding the feasibility of integrating the system into the workflow of trial staff were mixed. Overall, most of the interviewees (n=7) reported satisfaction with the system and expressed their desire to use it again in the future (n=8). However, some interviewees mentioned that they would only use the system for specific trials (n=2). Furthermore, 2 individuals highlighted that using the system resulted in labor savings during the recruitment process (n=2) and positively impacted recruitment numbers (n=1). The system was capable of reaching different groups of people compared to traditional recruitment methods, which, as a result, broadens the pool of potential patients (n=2).

### Evaluated Requirements

Requirements were derived from the statements of interviewees and are shown by frequency and category. We identified 4 requirements that were stated by 4 persons in the interviews. These were that whole patient numbers should be shown on the list to identify the patients properly. Further, they require a possibility to filter patients on the list after hospital wards. Two less concrete requirements were the better integration of the application in the clinical workflow and less false-positive suggestions on the list. All other requirements are shown in Multimedia Appendix 2. Multimedia Appendix 3 includes the code assignment for the requirements.

Five requirements were identified upon analyzing the interviews, which are highly similar to the ones in [11] (Multimedia Appendix 4). Three people mentioned the previously unimplemented requirements of integration into the local HIS and having flexible access to the lists. Although highlighting patients on the list is already possible, 1 interviewee proposed the ability to categorize and mark suggestions. This implies that the current implementation does not fully satisfy the users. One interviewee mentioned the need for more status options and an iterative patient search.

## Discussion

### Principal Findings

A comparison of the PRSs in question revealed that 8 (recruIT) and 11 (KAS+) of the 12 requirements identified in the previous analysis by Fitzer et al [11] can be fulfilled. Ten interviews were conducted with individuals involved in the recruitment of individuals for clinical trials at 3 distinct sites. Additionally, further requirements of the participants were identified. These requirements could be classified into different categories, and it was determined that integration into existing workflows is of particular importance for our interviewees. Many of the identified requirements are directly related to this.

### Degree of Compliance With Requirements

Although certain requirements outlined in [11] were not implemented in the systems under evaluation, none of the interviewees mentioned any of them. Based on the results of this study, it is assumed that both manually adding or removing patients from the list and implementing a sophisticated role and rights concept do not have a high priority for the interviewees. However, it cannot be determined whether these requirements would be useful in the context of the PRS. Given that this study is confined to a limited number of trial centers, it is possible that these requirements will only become relevant when more people are involved and multiple trials are supported.

### Interview Results

Certain interviewees mentioned new filtering options such as filtering for wards, despite the rarity or absence of these criteria in trial protocols. This indicates that for PRS implementation, official eligibility criteria alone might not be sufficient; additional filtering criteria that are specific to recruitment workflows may also be relevant. Further investigation may prove valuable in identifying other criteria that could enhance patient filtering.

Many of the mentioned parameters lead to diagnostic examinations which, taken together, occur often [20,21]. Diagnostic examinations can vary widely, and the resulting data that need to be queried by a PRS can vary as well. This can create challenges in collecting data from the local HIS. Access to high quality data from different clinical systems and electronic health records, which is an important part of a PRS, remains an unresolved issue and is the subject of ongoing research and development [22-24]. This finding was also reported by McCowan et al [7], who conducted stakeholder interviews for the project EHR4CR in 2015. Over half of the interviewees expressed the opinion that problems could arise from the lack of functionality in their HISs and the absence of crucial data items in the primary care systems [7]. Problems with filtering can arise from data that are documented unstructured, incorrect, or too late. As described in a data completeness analysis in 2022 [25], some data elements are found in less than 50% of electronic health records in German hospitals. Presumably for this reason, 1 participant mentioned that the data quality was not good enough.

Accuracy of suggestions is an area with several influencing factors such as the type of trial, general accessibility of the data, and data quality. One reason for false positives can be that not all of the important criteria are accessible, leading to suggestions that are technically correct, although the patient is still not eligible for the trial. The same result is achieved when there are fuzzy criteria, which need to be judged by trial staff. This is a problem also identified by Li et al [26] in 2021. They described that different scopes of research can lead to different definitions. In order to address this problem, we included the trial personnel in the definition of filter criteria. However, we learned that some criteria have to be checked manually, such as the cause of a disease or life expectancy [26]. Penberthy et al [27] also identified a high rate of false-positive suggestions in the evaluation of their PRS. They concluded that this was due to incomplete information about the patients, which prevented the exclusion criteria from being fully checked. However, some people have mentioned that few false positives are possible. Nevertheless, it is unclear whether this observation is based on concrete numbers or on the expectations of the participants.

The population examined in clinical studies is often criticized as not being representative. Older adults, women, and ethnic minorities in particular are less frequently included in clinical trials than they are represented in the general population [28-30]. Especially with the possibility to access a broader pool of patients, these tools could be used to face the underrepresentation of different groups. The ability of research staff to identify additional patients from diverse hospital wards is a phenomenon that Penberthy et al [27] also observed in their PRS evaluation. Additionally, including persons from other wards can happen more often to reach patients who are primarily treated for a different disease or health issue than that addressed in the trial.

Half of the participants were able to integrate the PRS in their daily routine, while others stated that this would not be fully possible. On closer examination of all statements of these persons, we could identify potential reasons for the missing integration and could see that 2 of these persons also criticized that pseudonymized lists are not practical. One of them stated that the lists should be generated earlier in the morning, and another demanded that the full patient names be included in the list. It is possible that integration could be easier if these issues are worked on. Despite the lack of comprehensive investigation into the PRS requirements, the integration of a PRS into existing systems, such as the official HIS, is a topic that is frequently discussed in various academic publications. In addition to the findings of Fitzer et al [11], Dugas et al [31] were able to derive this conclusion from a case study, while Schreiweis and Bergh [14] reached the same conclusion through stakeholder interviews.

### Features Already Implemented in KAS+

As mentioned above, the KAS+ system was not used with its full capabilities due to the study requirements. For each proposal, feedback was necessary, especially if it was marked as false positive—this was not possible with HIS integration. Therefore, this integration was not used for this study, which also disabled the connected features.

This is why some mentioned features are already implemented but have not been used, like the integration into HIS. Trial staff can access a screening list, which is constantly updated. Various filters can be applied to this screening list and electronic health records can be accessed directly from this list, provided that the user has sufficient rights. All suggestions are shown with a consent status that indicates, for example, whether they have signed an informed consent for this study, rejected, or withdrawn it.

### Implementation of Requirements

We could identify 32 requirements from the analyzed interviews. It is not possible to say which of the requirements are specific to a trial center or medical discipline and which are valid for a broader field of users. We assume that requirements mentioned by more than one person are at least not specific to one process. From all requirements, 12 were expressed in at least 2 interviews and are listed in Multimedia Appendix 4.

#### Adjustable Update Interval

Three of the requirements addressed the notification or update interval of the tool. By implementing adjustable intervals, all these requirements could be met. At least the features mentioned above should be available: weekly, daily, and real-time updates. It would be even better, especially with changing shift schedules, if the intervals could be chosen completely freely, that is, users could also specify certain weeks, days, or times when they want to receive notifications.

#### Integration in Workflow

Users want to be able to adapt the system to suit their needs, which correlates with the demand for a flexible PRS to be integrated into the daily workflow. This is particularly related to the demand for integration into the local HIS, which would also reduce system discontinuities in the solutions. The lack of integration sometimes leads to time-consuming workarounds, mainly caused by typing information from one system into the other. Reducing this work would therefore mean that the use of the screening list would take less time.

The tools should be embedded in the daily work of the trial centers. Trial staff work in a variety of workflows involving different groups of people, departments, and information sources [11,32]. In order to implement a working integration into existing processes, it is necessary to know them in detail. To the best of our knowledge, there is as yet no publicly described preliminary work on which to build [32]. For this reason, it makes sense to perform a complete workflow analysis before developing and implementing a PRS in order to avoid system discontinuities and other application issues. Furthermore, it is advisable to integrate the PRS directly into existing information systems when feasible.

#### Broad Filtering Options

The filtering options when generating the list have to cover criteria, which are relevant for the identification of eligible persons. Several studies have been conducted to find out which data elements are necessary to check all the eligibility criteria. Therefore, the criteria were bundled into data element groups. The studies that examined this issue list a broad range of data elements and their frequency, which can be used as a guide for the first implementation of a PRS [20,21]. Additionally, we could show that the filtering for wards and the multiple presence of parameters is necessary in the eyes of our participants. The PRS can only consider those data elements that are present in the clinical systems. However, there are data elements that are not routinely collected or are not of sufficient quality. As a primary requirement for the implementation of a PRS, it is therefore necessary that the system has access to a data pool that is as complete and up-to-date as possible. Nevertheless, eligibility criteria can be highly specialized. Thus, a more flexible approach where data elements can be extended continuously would be a way to face these issues.

#### Pseudonymization

The screening list should always show enough information to find the persons easily in HIS in order to keep the effort in locating the patients as low as possible. We consider this as the reason for full patient numbers or patient names to be shown on the list. Patient data should be pseudonymized for authorized users before being displayed on the screening list. A similar observation was made by Butte et al [33].

### Limitations

The main limitation of our study was the small number of participating trials: 10 trials at 3 different locations. Participants used a different PRS as already described above, and all locations had dissimilar local conditions, which might have had an impact on the results. The investigated trials varied in type, design, and the duration for which the trial staff used the PRS. Also, the way the interviews were documented varied slightly; while 2 of the included sites transcribed the interviews, 1 filled the form stenographically. Therefore, there is no additional information for the bounded questions for 1 trial. As we did only a qualitative analysis and used only our participants’ opinions regarding the false-positive and false-negative rates, we have no evidence that they always correlate with the quantitative numbers. Since the KAS+ test environment worked with daily generated PDF lists, many patients had already left the clinic when the trial staff checked the lists. Moreover, the KAS+ system would remove no longer suitable patients from the list, while this was not the case within the PDFs. This may have led to a higher false-positive rate.

### Conclusion

The trial staff had a high workload with the recruitment of patients. Especially in retrospective recruitment, where often hundreds of files of a ward have to be searched manually, the time required can be enormous and files of other wards are not even included. If a filter system such as recruIT or KAS+ succeeds in generating a list in which this number can be reduced, time can be saved, even if there are false-positive entries in this list. Although the evaluated PRS does not actually yet meet all requirements, all participants would use the system again, at least for certain trials, which shows the need of any kind of support.

Participants stated that, even with more accurate suggestions, a manual control is crucial, as there will always be discretionary criteria or other aspects that need a human judgment, which cannot be done by a PRS. The recruitment efficacy of the system can vary across different trials. Nonetheless, it remains to be seen. In any case, participants do not want a support system for each and every trial, in particular, if there are already well-established processes in place or if the identification of a test individual depends heavily on the doctor’s subjective assessment.

Our results are in line with test runs of comparable recruitment tools but also show that study personnel must be closely involved in the development to meet their needs like the filtering option for current wards or scheduled notifications. The next steps should be the exploration of the most needed parameters to increase the quality of the suggestions, the integration into HIS, and the implementation of an adjustable update and notification interval, as these are the most important aspects shown in this evaluation.

The future enhancement of the tools should be done in cooperation with the study personnel to create a tool that can easily be integrated into the workflow. To ensure this, future evaluations with a larger group of participants and a wider array of trials are necessary for a comprehensive analysis.

## Appendix

Multimedia Appendix 1 The full list of questions used as an interview guideline.

Multimedia Appendix 2 List of all the requirements derived from the qualitative analysis of interviews, ordered by the frequency of nomination.

Multimedia Appendix 3 List of all the requirements derived from qualitative analysis of interviews, ordered by category and shown with the code assignment for the requirements.

Multimedia Appendix 4 List of requirements defined by Fitzer et al [11] and implementation in the recruIT and KAS+ systems. Since a KAS+ test environment with different properties was used for this study, this is also indicated. In addition, the list shows how often the requirements were mentioned by the participants.

## Abbreviations

EHR4CR: electronic health records systems for clinical research
HIS: hospital information system
ICD: International Statistical Classification of Diseases
MIRACUM: Medical Informatics in Research and Care in University Medicine
OHDSI: Observational Health Data Sciences and Informatics
OMOP: Observational Medical Outcomes Partnership
PRS: patient recruitment system
