# Supplementary material for: User Requirements for an Electronic Patient Recruitment System: Semistructured Interview Analysis After First Implementation in 3 German University Hospitals
Source: JMIR Hum Factors. 2024 Sep 27;11:e56872. doi: 10.2196/56872 (PMC11470215; doi:10.2196/56872)
Supplement: Multimedia Appendix 1 [file humanfactors_v11i1e56872_app1.docx]

| **Demographic Data (all sites)** |  |
| --- | --- |
| age | free text |
| job title | multiple choice from:   - doctor - study coordinator - study nurse - medical documentary - study assistant - scientific assistant - technical personnel - other |
| other job title | free text |
| experience in patient recruitment in years | free text |
| **Interview questions (all sites)** |  |
| Please explain the recruitment process for this study without a system | free text |
| Please explain the recruitment process for this study with the system | free text |
| Were you able to integrate the system well into your recruitment processes and workflows? | yes/no |
| What were the main problems you had when using the system? | free text |
| What should have been different for the system to be (even) better integrated into the recruiting process? | free text |
| Is there anything else you would like to tell us about the system and your experience? | free text |
| Would you want to use the system again for other studies? | yes/no |
| If no, why not? | free text |
| **Additional questions (one site)** |  |
| How was the Recruitment support integrated in your recruitment Process (if not answered with the question before)? | free text |
| [Show an image of the pseudonymized version or the screening list]  Can you imagine to use such a system? | yes/no |
| How do you assess the complexity of the system? | Scale 1-5: 1= not complex 5= very complex |
| How would you rate the usability? | Scale 1-5: 1= easy 5= difficult |
| Is help from a technically skilled person needed / learned faster? | Scale 1-5: 1= makes no difference 5= would help noticeably |
| Were there many data inconsistencies? | Scale 1-5: 1= none 5= many |
| Have your expectations regarding the study participants been fulfilled? | fulfilled / not fulfilled / more than fulfilled / considerably more than fulfilled |
| Would you use this PRS more often? | [1-5] |
| Which frequency of suggestions would be optimal? | Live, daily, weekly, monthly, other |
| Have there been any changes within the study that might have an effect on the recruitment? | free text |
