# Supplementary figures and images for: User Requirements for an Electronic Patient Recruitment System: Semistructured Interview Analysis After First Implementation in 3 German University Hospitals
Source: JMIR Hum Factors. 2024 Sep 27;11:e56872. doi: 10.2196/56872 (PMC11470215; doi:10.2196/56872)

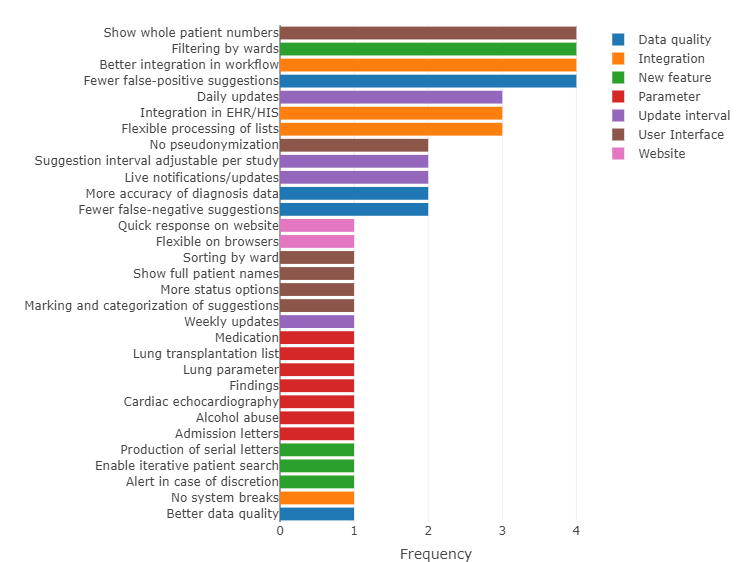

Supplement: Multimedia Appendix 2 [file humanfactors_v11i1e56872_app2.png]
