# Supplementary material for: User Requirements for an Electronic Patient Recruitment System: Semistructured Interview Analysis After First Implementation in 3 German University Hospitals
Source: JMIR Hum Factors. 2024 Sep 27;11:e56872. doi: 10.2196/56872 (PMC11470215; doi:10.2196/56872)
Supplement: Multimedia Appendix 3 [file humanfactors_v11i1e56872_app3.docx]

| requirement | group | n | Resulting from code (if divergent) |
| --- | --- | --- | --- |
|  |  |  |  |
| better data quality | Data quality | 1 | insufficient data quality |
| fewer false-negative suggestions | Data quality | 2 | Many false-negative suggestions |
| fewer false-positive suggestions | Data quality | 4 | Many false-positive suggestions |
| more accuracy of diagnosis data | Data quality | 2 | Inaccurency of diagnosis data (ICD) |
| better integration in workflow | Integration | 4 | bad integration in workflow |
| Flexible processing of lists | Integration | 3 | Flexible processing of lists |
| Integration in EHR/HIS | Integration | 3 | Integration in EHR/HIS |
| No system breaks | Integration | 1 | No system breaks |
| Alert in case of discretion | New feature | 1 | Alert in case of discretion |
| Enable iterative patient search | New feature | 1 | Enable iterative patient search |
| Filtering by wards | New feature | 4 | Filtering by wards |
| Production of serial letters | New feature | 1 | Production of serial letters |
| admission letters | Parameter | 1 | admission letters |
| alcohol abuse | Parameter | 1 | alcohol abuse |
| cardiac echocardiography | Parameter | 1 | cardiac echocardiography |
| findings | Parameter | 1 | findings |
| lung parameter | Parameter | 1 | lung parameter |
| lung transplantation list | Parameter | 1 | lung transplantation list |
| medication | Parameter | 1 | medication |
| Daily updates | Update interval | 3 | Daily updates |
| Live notifications/updates | Update interval | 2 | Live notifications/updates |
| Suggestion interval adjustable per study | Update interval | 2 | Suggestion interval adjustable per study |
| Weekly updates | Update interval | 1 | Weekly Updates/Notifications |
| Marking and categorization of suggestions | User Interface | 1 | Marking and categorization of suggestions |
| More status options | User Interface | 1 | More status options |
| no pseudonymization | User Interface | 2 | Pseudonymized lists are not practical |
| show full patient names | User Interface | 1 | show full patient names |
| show whole patient numbers | User Interface | 4 | show whole patient numbers |
| sorting by ward | User Interface | 1 | sorting by ward |
| Flexible on browsers | Website | 1 | Flexible on browsers |
| quick response on website | Website | 1 | quick response on website |
