# Supplementary material for: User Requirements for an Electronic Patient Recruitment System: Semistructured Interview Analysis After First Implementation in 3 German University Hospitals
Source: JMIR Hum Factors. 2024 Sep 27;11:e56872. doi: 10.2196/56872 (PMC11470215; doi:10.2196/56872)
Supplement: Multimedia Appendix 4 [file humanfactors_v11i1e56872_app4.docx]

|  | **Requirement by Fitzer et al [10]** | **recruIT** | **KAS+ Evaluation Environment** | **KAS+** | **Requirements identified in this paper** | **n** |
| --- | --- | --- | --- | --- | --- | --- |
| **Notifications** |  |  |  |  |  |  |
|  | users are instantly notified if new suggestions are available | 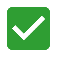 | 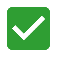 | 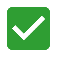 |  |  |
|  | notifications are adjustable to individual preferences by the user | 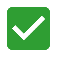 | 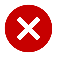 | 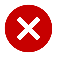 | Adjustable Update Interval | 3 |
|  |  | 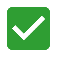 | 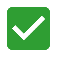 | 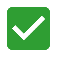 | Daily Updates | 4 |
|  |  | 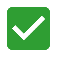 | 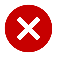 | 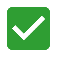 | Live Updates | 3 |
| **Overview of patients** |  |  |  |  |  |  |
|  | supports a list of all patient suggestions | 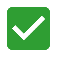 | 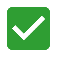 | 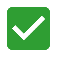 |  |  |
|  | possibility to check suggestions by themselves | 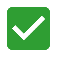 | 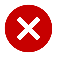 | 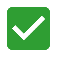 | Flexible processing of lists | 3 |
|  | the list with suggestions is integrated into existing systems | 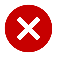 | 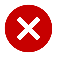 | 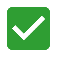 | Integration in HIS | 3 |
|  | option to mark participants | 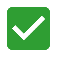 | 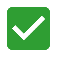 | 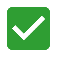 | Marking and categorization of suggestions | 1 |
|  | option to make notes | 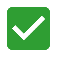 | 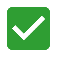 | 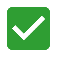 |  |  |
|  | option to track the recruitment status | 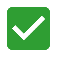 | 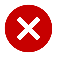 | 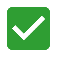 | More status options | 1 |
|  | Edit recruitment list by |  |  |  |  |  |
|  | manually add patients | 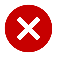 | 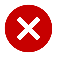 | 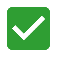 |  |  |
|  | remove patients | 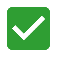 | 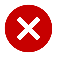 | 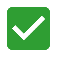 |  |  |
|  | patient summaries are integrated into PRS | 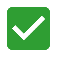 | 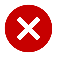 | 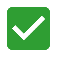 |  |  |
|  |  | 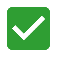 | 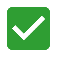 | 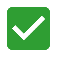 | no pseudonymization | 6 |
|  |  | 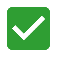 | 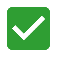 | 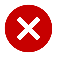 | show whole Patient Number | 4 |
| **Search** |  |  |  |  |  |  |
|  | offers sophisticated search options | 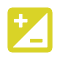 | 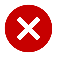 | 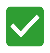 | Enable iterative patient search | 1 |
|  |  | 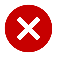 | 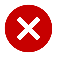 | 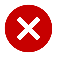 | filtering by wards | 5 |
|  |  | 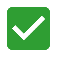 | 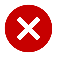 | 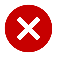 | Filtering by multiple presence of parameters | 2 |
| **User Management and Interface** |  |  |  |  |  |  |
|  | contain a sophisticated rights concept to account for the various roles in the trial and at the clinical center | 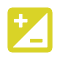 | 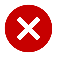 | 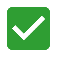 |  |  |

(
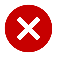
= not implemented,
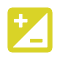
= partially implemented,
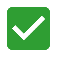
=implemented)
